# Peroral cholangioscopy-guided laser lithotripsy using a thin cholangioscope under balloon enteroscopy for Roux-en-Y anastomosis

**DOI:** 10.1055/a-2738-7298

**Published:** 2025-11-19

**Authors:** Yuki Tanisaka, Shomei Ryozawa, Masafumi Mizuide, Akashi Fujita, Ryuichi Watanabe, Ryosuke Hamamura

**Affiliations:** 1 183786 Department of Gastroenterology, Saitama Medical University International Medical Center, Hidaka, Japan

Although balloon enteroscopy is helpful, stone extraction in patients with Roux-en-Y anastomosis can be very difficult, particularly in cases with multiple large stones 1 2 . Recently, a thin cholangioscope (eyeMAX; Micro-Tech, China) with a length of 219 cm and a diameter of 9-Fr ( Fig. 1 ) has enabled peroral cholangioscopy (POCS)-guided interventions to be performed under balloon enteroscopy 3 . POCS-guided intraductal interventions using either laser lithotripsy or electrohydraulic lithotripsy (EHL) can facilitate the extraction of difficult stones 4 5 . It has been reported that, compared with EHL, laser lithotripsy provides the advantage of more precise stone targeting, thereby reducing the risk of injury to the surrounding tissue 3 . We report a case of successful laser lithotripsy using a thin cholangioscope under balloon enteroscopy in a patient with Roux-en-Y anastomosis.

**Fig. 1:**
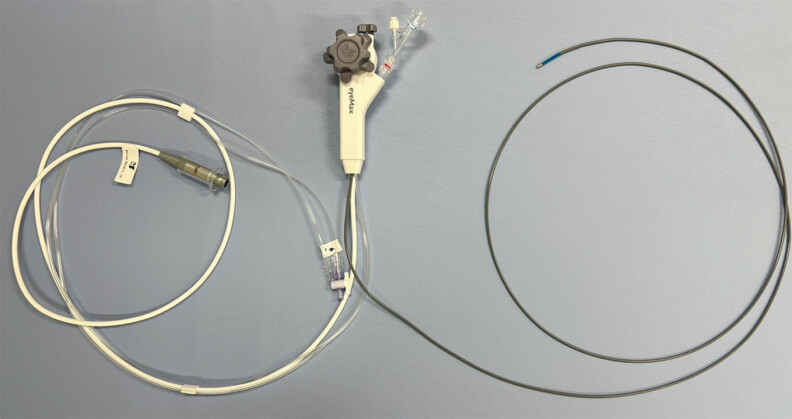
A thin cholangioscope (eyeMAX; Micro-Tech, China) measuring 219 cm in length, with a diameter of 9-Fr.

A 61-year-old woman with multiple large stones in the right intrahepatic bile duct, who had previously undergone hepaticojejunostomy with Roux-en-Y, was referred to us ( Fig. 2 ). Endoscopic retrograde cholangiopancreatography was performed using a short-type single-balloon enteroscopy (SIF-H290; Olympus Marketing, Japan) with a working length of 152 cm and a working channel of 3.2 mm in diameter ( Video 1 1 2 ). Cholangiography revealed multiple large stones in the right intrahepatic bile duct ( Fig. 3 ). Subsequently, POCS was performed using a thin cholangioscope and revealed multiple large stones in the intrahepatic bile duct ( Fig. 4 **a** ). A laser fiber was introduced, and POCS-guided laser lithotripsy was performed under a clear field of view. Precise targeting and fragmentation of the stones were achieved safely ( Fig. 4 **b,c** ), resulting in complete stone extraction ( Fig. 5 ).

**Fig. 2:**
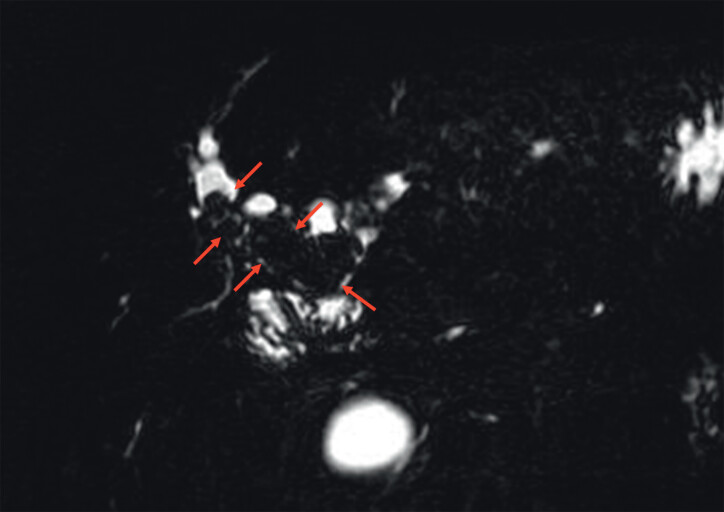
Magnetic resonance imaging revealing multiple large stones in the right intrahepatic bile duct (red arrow).

Peroral cholangioscopy-guided laser lithotripsy using a thin cholangioscope under balloon enteroscopy for Roux-en-Y anastomosis.Video 1

**Fig. 3:**
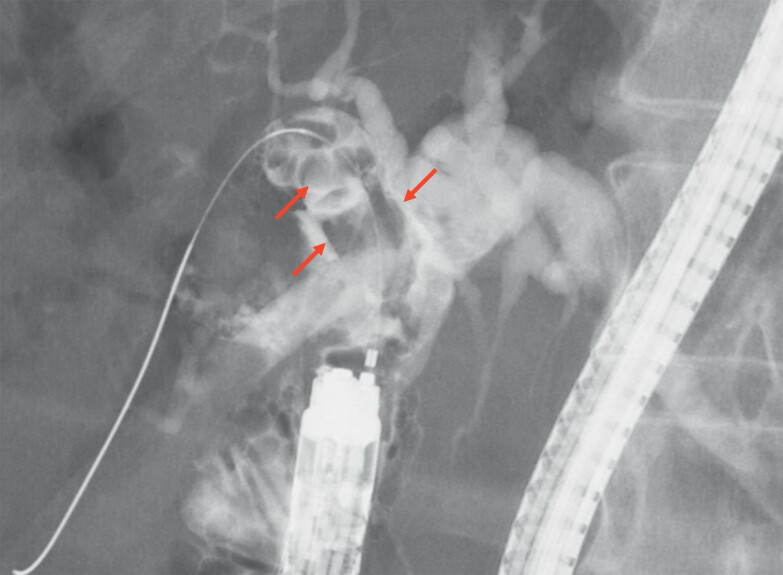
Cholangiography revealing multiple large stones in the right intrahepatic bile duct (red arrow).

**Fig. 4:**
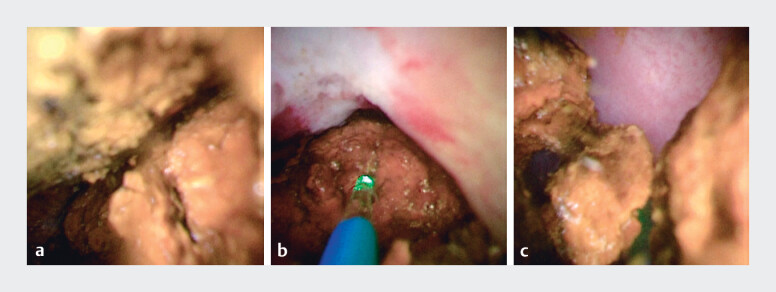
Cholangioscopy findings. **a** Cholangioscopy revealing multiple large stones in the right intrahepatic bile duct. **b, c** Precise targeting and fragmentation of the stones were achieved safely.

**Fig. 5:**
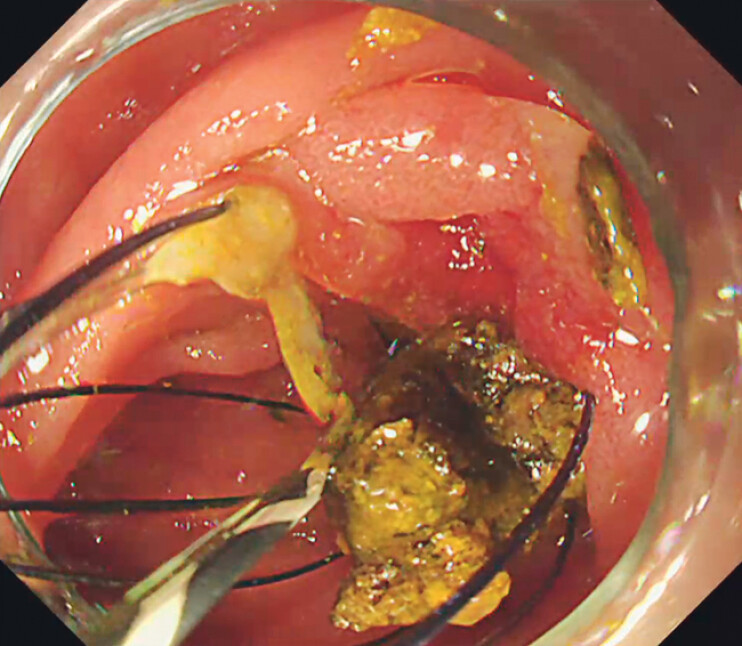
Endoscopic findings revealing successful complete stone extraction.

This case highlights the utility of POCS-guided laser lithotripsy using a thin cholangioscope, even in the setting of balloon enteroscopy. Both the thin cholangioscope and the laser fiber can improve the success rate of stone extraction in such patients.

Endoscopy_UCTN_Code_TTT_1AR_2AH

## Notes

Endoscopy E-Videos https://eref.thieme.de/e-videos
*E-Videos* is an open access online section of the journal *Endoscopy* , reporting on interesting cases and new techniques in gastroenterological endoscopy. All papers include a high-quality video and are published with a Creative Commons CC-BY license. Endoscopy E-Videos qualify for HINARI discounts and waivers and eligibility is automatically checked during the submission process. We grant 100% waivers to articles whose corresponding authors are based in Group A countries and 50% waivers to those who are based in Group B countries as classified by Research4Life (see: https://www.research4life.org/access/eligibility/ ). This section has its own submission website at https://mc.manuscriptcentral.com/e-videos .
